# Gender-specific changes in vision-related quality of life over time – results from the population-based Gutenberg Health Study

**DOI:** 10.1007/s00417-025-06741-9

**Published:** 2025-02-11

**Authors:** Alica Hartmann, Stephanie D. Grabitz, Philipp S. Wild, Karl J. Lackner, Thomas Münzel, Jasmin Ghaemi Kerahrodi, Susanne Singer, Katharina Geschke, Jörn M. Schattenberg, Stavros Konstantinides, Norbert Pfeiffer, Alexander K. Schuster

**Affiliations:** 1 Department of Ophthalmology, University Medical Center of the Johannes Gutenberg-University Mainz, Langenbeckstraße 1, 55131 Mainz, Germany; 2 Preventive Cardiology and Preventive Medicine – Department of Cardiology, University Medical Center Mainz, Mainz, Germany; 3 Center for Thrombosis and Hemostasis, University Medical Center Mainz, Mainz, Germany; 4 German Center for Cardiovascular Research (DZHK), Partner Site Rhine-Main, Mainz, Germany; 5 Institute of Molecular Biology (IMB), Mainz, Germany; 6 Institute of Clinical Chemistry and Laboratory Medicine, University Medical Center of the Johannes Gutenberg-University Mainz, Mainz, Germany; 7 Center for Cardiology --- Cardiology I, University Medical Center of the Johannes Gutenberg-University Mainz, Mainz, Germany; 8 Department of Psychosomatic Medicine and Psychotherapy, University Medical Center of the Johannes Gutenberg University Mainz, Mainz, Germany; 9 Division of Epidemiology and Health Services Research, Institute of Medical Biostatistics, Epidemiology and Informatics (IMBEI), University Medical Center of the Johannes Gutenberg University Mainz, Mainz, Germany; 10 Metabolic Liver Research Center and Medicine, University Medical Center, Mainz, Germany; 11 University Cancer Center of Johannes Gutenberg-University Mainz, Mainz, Germany; 12 Partner site Frankfurt-Mainz, German Cancer Consortium (DKTK), Mainz, Germany; 13 Department of Psychiatry and Psychotherapy, University Medical Center of the Johannes Gutenberg-University Mainz, Mainz, Germany

**Keywords:** Quality of life, Visual acuity, Visual impairment, Gender factors, Healthy ageing, VFQ-25

## Abstract

**Purpose:**

To investigate potential gender- and age-specific changes over time in vision-related quality of life (VRQoL) on a population-based level. Further, factors associated with changes in VRQoL will be explored.

**Methods:**

The Gutenberg Health Study is a population-based, prospective, observational, single-center cohort study in Germany. VRQoL was quantified at baseline and 5-year follow-up using the visual function scale (VFS) and socio-emotional scale (SES-VRQoL). VFS and SES-VRQoL are calculated using the “National Eye Institute 25-Item Visual Functioning Questionnaire” (NEI-VFQ-25). Both scales range from 0 to 100, 0 corresponds to the sum that would be achieved if a participant had answered all items with the worst performance, and 100 corresponds to the sum of all items answered with the best possible performance. Distance-corrected visual acuity was measured in both eyes. Univariable and multivariable linear regression analyses were conducted to identify ophthalmic and sociodemographic predictors of VRQoL.

**Results:**

A total of 10,152 participants (mean age 54.2 years; 49.2% female) were included in the analysis. The mean visual functioning decreased from 89.6 (IQR: 81.3, 95.1) at baseline to 85.9 (IQR: 79.2, 92.6) at 5-year follow-up in the VFS (*p* < 0.001). Participants' socio-emotional well-being remained the same from baseline to 5-year follow-up in the SES-VRQoL. In multivariable linear regression analysis, older age (0.03, *p* = 0.002) and female gender (-1.00, *p* < 0.001) were associated with a VFS change. Higher baseline socioeconomic status was associated with a slightly positive increase in VFS (0.07, *p* = 0.001). Deterioration of visual acuity in the better and worse-seeing eye was associated with negative VFS change over 5 years (better-seeing eye: -5.41, *p* < 0.001, worse-seeing eye: -7.35, *p* < 0.001). Baseline socioeconomic status was associated with SES-VRQoL change (0.06, *p* < 0.001). The negative change in visual acuity showed an association with negative SES-VRQoL in the better (-4.15, *p* < 0.001) and worse-seeing eye (-3.75, *p* < 0.001). Stratification of the regression models by age and gender showed greater reductions in VFS scores with visual acuity changes in participants aged 65 years or older and a more pronounced decrease in female participants over 5 years.

**Conclusions:**

This study demonstrated an association between visual acuity change and change in VRQoL over 5 years, with a greater decrease in female participants and participants aged 65 years or older. The better-seeing eye and the worse-seeing eye both had an impact on changes in VRQoL.

**Key messages:**

***What is known***

- Previous studies have predominantly used cross-sectional designs to investigate the correlation between visual acuity and vision-related quality of life, with limited insights into how visual acuity changes over time affect vision-related quality of life in a large cohort.

***What is new***

- This study demonstrates that visual acuity changes significantly impact VRQoL over a 5-year period, with a notable decrease observed in female participants and those aged 65 years or older.
- Analysis reveals both the better-seeing and worse-seeing eye contribute to changes in VRQoL, highlighting the necessity of comprehensive visual assessments in both eyes for a more accurate understanding of VRQoL outcomes.

**Supplementary Information:**

The online version contains supplementary material available at 10.1007/s00417-025-06741-9.

## Introduction

Subjective perception of difficulties due to vision and their impact on daily life activities are important when assessing the burden of ophthalmic diseases. Patient-reported outcomes analyzing vision-related functioning and quality of life are gaining importance in evaluating therapeutic interventions and are included as primary outcomes in clinical studies. Vision-related quality of life (VRQoL) describes how vision affects the life of a person and their satisfaction with vision ability [1].

To date, VRQoL has been mostly examined in cross-sectional studies. These cross-sectional studies showed worse VRQoL in the presence of visual impairment (VI) compared to no VI [1–3]. Several studies have demonstrated that loss of visual functioning and VI is associated with a decline in VRQoL [3–5]. VI can lead to emotional or physical difficulties resulting in limitations in social life [6]. In addition, higher age and lower socioeconomic status is associated with lower VRQoL [7, 8].

Few studies have examined gender-related differences in VRQoL in cross-sectional studies: one study found slightly worse VRQoL in women [9]. The Gutenberg Health Study (GHS) has previously reported gender-specific differences in VRQoL in a cross-sectional approach with worse VRQoL in women [3, 10]. When examining gender-related differences in health-related quality of life (HRQoL), numerous studies consistently report lower scores for women [11–14]. A study from the United States, which analyzed four national datasets, found that women had worse HRQoL outcomes compared to men, with sociodemographic and socioeconomic disparities partially accounting for these differences [13]. Similarly, another study demonstrated that women had significantly worse HRQoL than men across all subscales, even after adjusting for factors such as age and chronic conditions [11].

However, it is unknown to date whether the course of VRQoL over time also differs between genders. Possible gender differences could be due to a higher prevalence of blindness in women [15], a difference in socioeconomic status [16] or different symptom reporting [17].

Several studies found that VRQoL decreases with age [1, 8, 18]. Thus, it may be important to implement screening measures early to detect VI and counteract a decrease in VRQoL early on [8]. Concerning visual acuity, both the better-seeing and worse-seeing eye have an influence on VRQoL: the better-seeing eye has a stronger influence and the worse eye has a minor influence on the VRQoL, thus the function of both eyes should also be included in clinical decision-making processes [3].

McKean-Cowdin et al. examined changes in visual acuity and HRQoL in a population-based cohort study with 3,169 individuals in La Puente, California (age: 40 years or older). A decreased visual acuity over 4 years was linked to decreased visual functionality and overall well-being [19].

Cross-sectional studies reported that individuals with lower socioeconomic status also have worse VRQoL [20, 21]. It may be important to provide more counseling and information to these individuals in the clinical context to increase awareness of eye disease. It is also important to reach this group of people in prevention programs [20, 21].

This study focuses on changes in VRQoL over time and evaluates whether there are gender-, age- or socioeconomic-specific differences in this regard on a population-based level. Further, the association between the change in VRQoL with the change in visual acuity of the better and worse-seeing eye is evaluated.

## Method

### Study sample

The GHS is a population-based, prospective, observational, single-center cohort study in the Rhine-Main-Region in Germany. The sampling was stratified for gender, residence (urban or rural) and age-decade. At baseline, 15,010 individuals were included (in the years 2007–2012) and 12,423 of them were re-examined after 5 years (2012–2017). The inclusion criteria for the current analysis were the availability of NEI-VFQ-25 (National Eye Institute Visual Function Questionnaire) scores at baseline and at 5-year follow-up to be able to calculate the change of VFQ-25 scores for each study participant. This reduced the number of subjects to 10,152 participants.

### Ophthalmic parameters

Objective refraction and distance-corrected visual acuity were measured in both eyes with Humphrey Automated Refractor/Keratometer at baseline and 5-year follow-up. Distance corrected visual acuity was measured with built-in Snellen charts, ranging from 20/400 to 40/20. The values were transformed to LogMar for statistical analysis. Spherical equivalent was measured through the following calculation: spherical equivalent value plus half of the cylindrical power [3]. A short interview was conducted before the eye examination to assess a potential history of eye disease.

The WHO definition of VI was used to categorize visual acuity into no, mild, and moderate/severe vision impairment groups.

### Quality of life and socioeconomic status

Vision-related quality of life was assessed using the NEI-VFQ-25 questionnaire [22]. The questionnaire was completed using both eyes and reading glasses if needed. Socioeconomic status was calculated according to Lampert and Kroll [23], this score consists of the following factors: highest education (school/vocational training), position in occupation, and income. With regard to occupation, participants were asked what occupation was currently held or, in the case of unemployment/retirement, what occupation was previously held. The survey also asked for a classification of the occupation into an occupational group (self-employed, academics in the liberal professions such as doctors, lawyers, officials, employees, trainees, or assisting family members). Regarding income, the survey asked about the households' net monthly income. The household size was considered and the net income was adjusted for the individual person. Status was defined as a socioeconomic status range between 3 (lowest) and 21 (highest) socioeconomic status [23].

### Statistical analysis

A descriptive analysis was conducted and stratified by gender to examine potential gender differences at baseline time point. For categorical parameters, absolute and relative frequencies were computed. For continuous variables, mean and standard deviation were calculated for approximately normal-distributed data, otherwise median and interquartile range. We calculated the “visual functioning scale (VFS)” and “socioemotional scale (SES-VRQoL)”. This is relevant to counteract limitations of the questionnaire (e.g. influenced by multidimensionality) [24]. Scales were based on Rasch-transformed individual-level NEI-VFQ-25 data, as used in several other studies [25, 26]. The questionnaire is composed of 25 questions and includes 12 subscales: general health, general vision, ocular pain, near activities, distance activities, social functioning, mental health, role difficulties, dependency, driving, color vision, and peripheral vision. The VFS and SES-VRQoL were calculated based on the principal component analysis approach of Pseudovs. et al. [24]. Both scales were then converted to a 0–100 point scale, where 0 represents the sum a participant would achieve if all items were answered with the worst possible performance, and 100 represents the sum for the best possible performance. This conversion to a 0–100 scale was done to ensure comparability with other studies.

We investigated the factors associated with changes in the VFS over a five-year period. The analysis included several independent variables, namely gender (reference: male participants), age, baseline VFS, socioeconomic status, and visual acuity in both the better- and worse-seeing eye. Both univariable and multivariable linear regression models were employed to assess how each factor is related to changes in VFS, both individually and while controlling for other variables.

In a separate linear regression analysis, the same independent variables were used to examine their influence on changes in the SES-VRQoL.

We created additional models with stratification by age group (< 65 years versus ≥ 65 years). Finally, the models were calculated separated for women and men to examine gender-related differences and additionally a gender specific interaction analysis was conducted.

Data were processed with the statistical program R (version: 4.0.3 (2020–10-10)).

## Results

The mean age of the study population was 54.2 ± 10.8 years at baseline, and 49.2% were female (Table 1). VFS was slightly higher in men than in women at baseline (89.59 vs. 89.56).

**Table 1:** Participants’ characteristics (*N* = 10,152, baseline) and quality of life parameters. Data from the German population-based Gutenberg Health Study (2007–2012)

	Overall	Men	Women
	10,152	5161	4991
Age [y], mean (SD)	54.2 (10.8)	54.5 (10.9)	53.8 (10.7)
Age categories, n (%)			
35–44	2349 (23.1)	1144 (22.2)	1205 (24.1)
45–54	2863 (28.2)	1438 (27.9)	1425 (28.6)
55–64	2734 (26.9)	1386 (26.9)	1348 (27.0)
65–74	2206 (21.7)	1193 (23.1)	1013 (20.3)
Ophthalmic parameters			
Visual acuity [logMar], better eye, mean (SD)	0.03 (0.09)	0.02 (0.09)	0.04 (0.09)
Visual acuity [logMar], worse eye, mean (SD)	0.11 (0.22)	0.10 (0.22)	0.12 (0.22)
Spherical equivalent [diopters], right eye, mean (SD)	−0.50 (2.49)	−0.53 (2.42)	−0.47 (2.57)
Spherical equivalent [diopters], left eye, mean (SD)	−0.49 (2.50)	−0.53 (2.45)	−0.45 (2.55)
Glaucoma (ISGEO definition), n (%)	72 (0.9)	41 (1.0)	31 (0.8)
History of eye surgery, n (%)	692 (6.8)	326 (6.3)	366 (7.3)
General health and socioeconomic status			
Arterial hypertension, n (%)	4792 (47.2)	2719 (52.7)	2073 (41.6)
BMI [kg/m^2^], mean (SD)	27.1 (4.8)	27.7 (4.2)	26.4 (5.3)
Socioeconomic status, mean (SD)	13.5 (4.3)	14.2 (4.4)	12.7 (4.1)
Vision-related quality of life			
Visual functioning scale, median [IQR]	89.57 [81.30, 95.10]	89.59 [84.02, 95.10]	89.56 [81.25, 94.49]
Socioemotional scale, median [IQR]	100 [95.07, 100.00]	100 [94.62, 100.00]	100 [95.07, 100.00]

*y* years, *SD* standard deviation

**Fig. 1:**
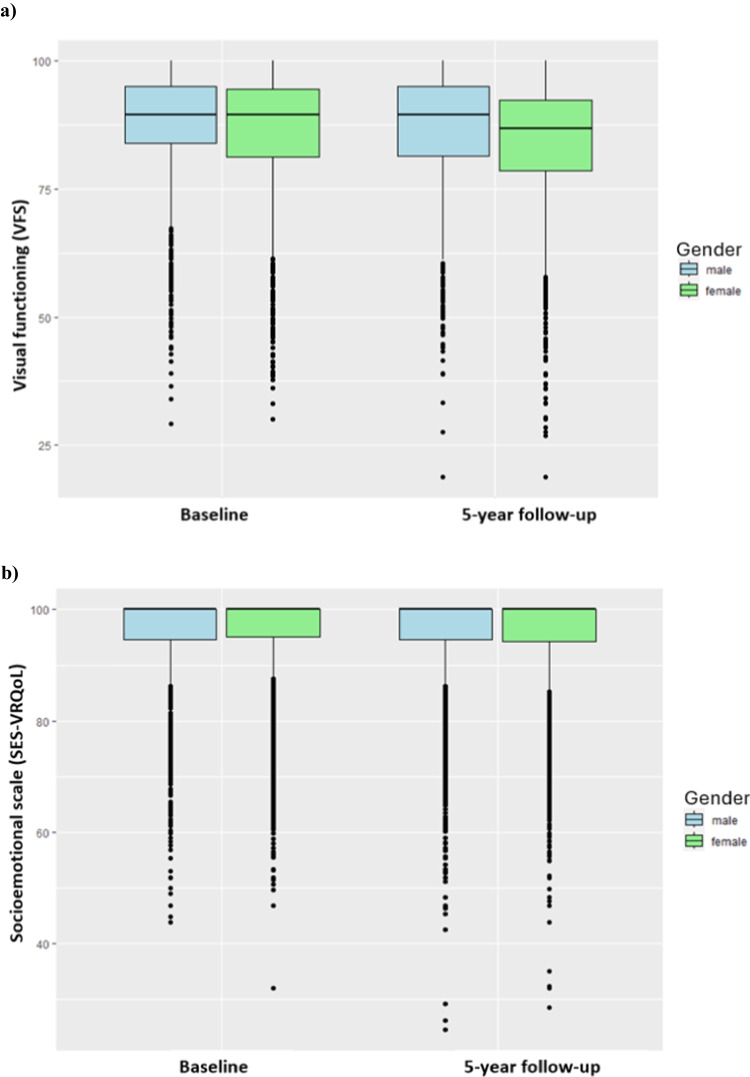
**a** Visual functioning scale (VFS) at baseline and 5-year follow-up stratified by gender and **b**) socioemotional scale (SES-VRQoL) at baseline and 5-year follow-up stratified by gender. Data from the German population-based Gutenberg Health Study (2007–2017)

During the 5 years, visual acuity decreased by 0.05 LogMAR in women and by 0.03 LogMAR in men. VFS decreased more in women (−1.3) than in men (−0.9; *p* = 0.05) (Fig. 1a). SES-VRQoL scores changed barely over 5 years for both men and women (Fig. 1b). Socioeconomic status decreased overall by −0.19 (SD: 1.93), and decreased significantly more for women (−0.26 (SD: 1.96) vs. −0.12 (SD: 1.90)). Over the period from baseline to the five-year follow-up, the number of eyes with VI increased (Table S1).

**Fig. 2:**
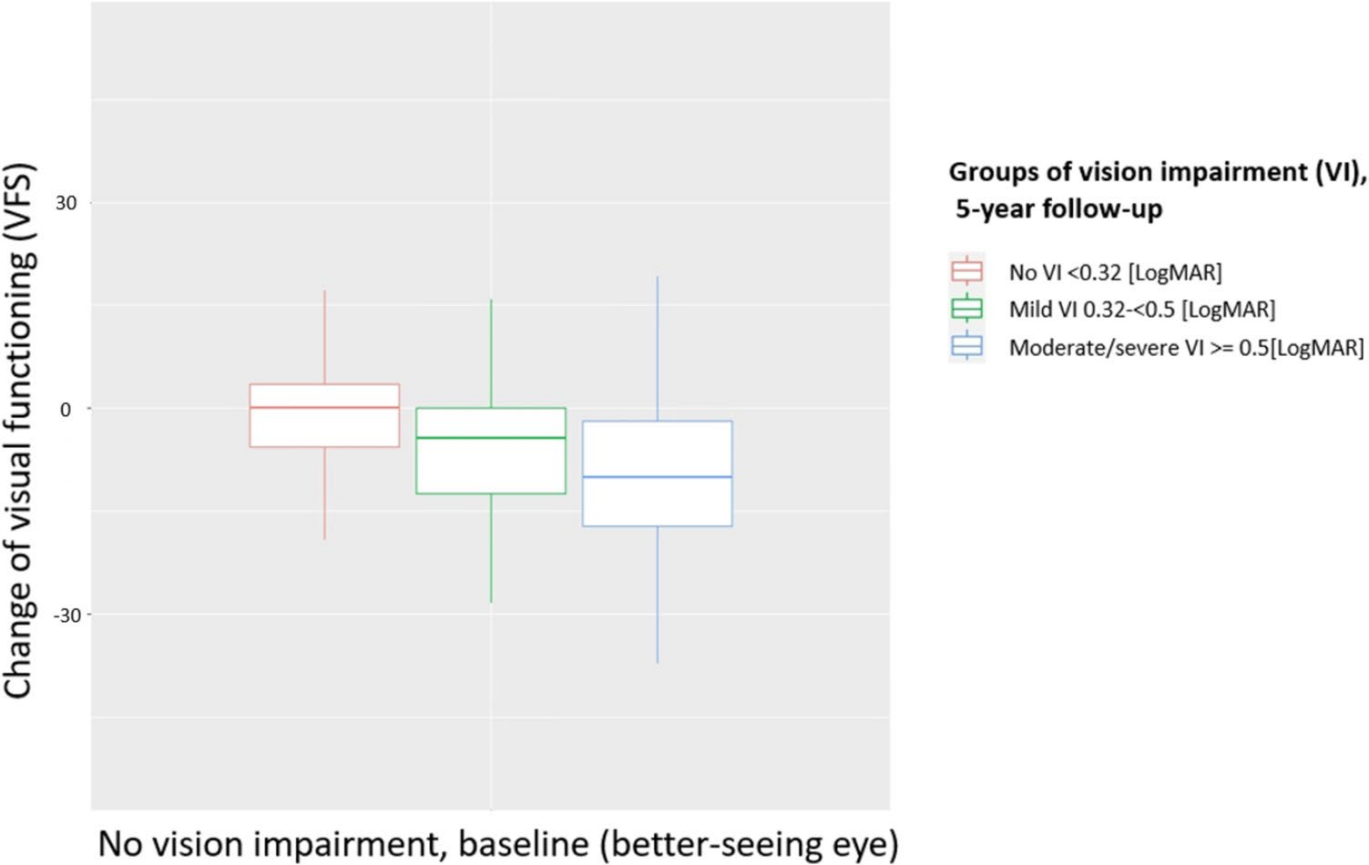
Change of visual functioning scale (VFS) over 5 years with no vision impairment at baseline (better-seeing eye, (*n* = 9,875)). Grouped into the follow-up vision impairment groups of the better-seeing eye (no (*n* = 8,997), mild (*n* = 83), and moderate/severe (*n* = 51)). Results from the population-based Gutenberg Health Study (2007–2017)

Figure 2 shows the change in VFS over five years for participants with no VI at baseline. In participants with no VI over time, VFS decreased slightly, while a change from no VI at baseline to mild VI at a 5-year follow-up was associated with a more significant decrease in VFS, which was even more pronounced with a change from no VI at baseline to moderate/severe VI over the 5 years.

Table 2 illustrates how changes in VI status over five years influence the VFS scores of the study participants. Specifically, it categorizes individuals based on their VI status in the better-seeing eye at baseline and at the five-year follow-up. The table shows how VFS scores change depending on whether participants’ vision remained stable, improved, or worsened over this period. Participants who consistently had no VI in the better-seeing eye over 5 years showed a slight worsening of VFS over five years by −1.08 VFS score points. Moreover, those with no VI in the better-seeing eye at baseline and a change to mild or moderate VI at 5-year follow-up had decreasing VFS scores. Participants whose vision worsened from mild VI at baseline to moderate/severe VI at follow-up experienced a small improvement in VFS scores (+ 0.83 points). Study participants with improvement in VI categories in the better-seeing eye showed improvement in VFS scores. Participants who initially had moderate/severe VI in the better-seeing eye at baseline and an improvement to no VI at 5-year follow-up showed an increase in VFS scores of 14.8 points (Table 2), however, this VI change in the better-seeing eye was only the case in 9 participants. Supplemental Fig. 1 demonstrates the change in visual acuity (LogMAR) and the change in the VFS over five years for the better-seeing eye (a) and the worse-seeing eye (b). In both cases, a negative association is evident, indicating that a decline in visual acuity corresponds with a decrease in VFS. Outliers (1.31%, *n* = 118) were identified, showing no visual acuity decline greater than 0.1 LogMAR in either eye over five years, a change not considered clinically relevant [27], yet experiencing a high deterioration in VFS of more than 15 points, which normally corresponds with a significant decline in visual acutiy [28]. At baseline, this group reported better general health, but a significantly worse general health status was observed at follow-up. Additionally, the group was significantly younger, had a higher proportion of female participants, and showed a greater decline in SES-VRQoL (Suppl. Table 2).

**Table 2:** Change of NEI-VFQ-25 visual functioning scale (VFS) scores by categories of vision impairment (VI) at baseline and 5-year follow-up (better-seeing eye) of the German population-based Gutenberg Health Study (GHS), time frame of 2007–2017

	Better-seeing eye, 5-year follow-up			
	Change of VFS over 5 years	No VI (*n* = 8,997)	Mild VI (*n* = 83)	Moderate/severe VI (*n* = 51)
Better-seeing eye, Baseline	No VI (*n* = 9,875)	−1.08 points	−5.17 points	−8.96 points
	Mild VI (*n* = 28)	+ 14.79 points	−5.53 points	+ 0.83 points
	Moderate/severe VI (*n* = 32)	+ 14.76 points	+ 4.72 points	−5.45 points

*VFS* visual functioning scale

### Linear regression analysis

Gender and age were associated with VFS change, with a higher VFS reduction in female participants compared to male participants (Table 3). Baseline socioeconomic status was associated with change in VFS but change in socioeconomic status was not associated with change in VFS.

**Table 3:** Association analysis between gender, age, visual acuity (in the better-/worse-seeing eye) and socioeconomic status with the change in the visual functioning scale (VFS) over 5 years. Data from the German population-based Gutenberg Health Study (2007–2017)

	Univariable			Multivariable		
Dependent variable: Change of VFS	B	95%-CI	p-value	B	95%-CI	p-value
Gender, female						
Baseline	−0.37	[−0.74–0.01]	0.05	−1.00	[−1.35- −0.64]	< 0.001
Age						
Baseline	0.07	[0.05–0.09]	< 0.001	0.03	[0.01–0.05]	0.002
VFS						
Baseline	−0.41	[−0.43- −0.40]	< 0.001	−0.45	[−0.47- −0.43]	< 0.001
Socioeconomic status						
Baseline	−0.01	[−0.06–0.03]	0.56	0.07	[0.03–0.11]	0.001
5-year change	−0.20	[−0.30- −0.11]	< 0.001	−0.06	[−0.15–0.03]	0.22
Visual acuity, better-seeing eye						
Baseline	11.30	[9.22–13.39]	< 0.001	−3.16	[−5.69- −0.63]	0.01
5-year change	−5.97	[−7.21–4.72]	< 0.001	−5.41	[−6.69- −4.13]	< 0.001
Visual acuity, worse-seeing eye						
Baseline	3.22	[2.38–4.05]	< 0.001	−3.41	[−4.52- −2.29]	< 0.001
5-year change	−10.88	[−12.53- −9.23]	< 0.001	−7.35	[−9.18- −5.52]	< 0.001

*VFS* Visual functioning scale; reference group for gender is male participants

The baseline visual acuity in the better-seeing and worse-seeing eye, both were univariable positively associated with VFS change over 5 years due to collinearity with lower VFS at baseline. In the multivariable regression model, there was a negative association for the change of VFS with associated with the change of visual acuity in the better- and worse-seeing eye over 5 years.

Additionally, associations between the change of SES-VRQoL with visual acuity (baseline/change) and socioeconomic status (baseline/change) were evaluated in multivariable regression analysis (Table 4). Female gender and age were not associated with the SES-VRQoL. Baseline socioeconomic status was positively associated with the change in SES-VRQoL scores, while change in socioeconomic status over 5 years was not associated in the multivariable model. Baseline and changes in visual acuity were associated with a reduction in SES-VRQoL scores over 5 years.

**Table 4:** Association analysis between gender, age, visual acuity (in the better-/worse-seeing eye) and socioeconomic status with a change in socioemotional scale (SES-VRQoL) over 5 years. Data from the German population-based Gutenberg Health Study (2007–2017)

	Univariable			Multivariable		
Dependent variable: Change of SES-VRQoL	B	95%-CI	p-value	B	95%-CI	p-value
Gender, female						
Baseline	−0.24	[−0.51–0.04]	0.09	0.04	[−0.22–0.30]	0.77
Age						
Baseline	−0.01	[−0.02–0.01	0.31	−0.01	[−0.02–0.01]	0.31
SES-VRQoL						
Baseline	0.06	[0.03–0.09]	< 0.001	−0.48	[−0.50- −0.46]	< 0.001
Socioeconomic status						
Baseline	0.06	[0.03–0.09]	< 0.001	0.06	[0.03–0.10]	< 0.001
5-year change	−0.08	[−0.15- −0.01]	0.02	−0.03	[−0.10–0.04]	0.43
Visual acuity, better-seeing eye						
Baseline	−0.46	[−2.03–1.11]	0.56	−7.21	[−9.08- −5.35]	< 0.001
5-year change	−3.96	[−4.92- −3.00]	< 0.001	−4.15	[−5.14- −3.16	< 0.001
Visual acuity, worse-seeing eye						
Baseline	0.70	[0.06–1.33]	0.03	−1.41	[−2.24–0.58]	< 0.001
5-year change	−5.38	[−6.61- −4.16]	< 0.001	−3.75	[−5.11- −2.39]	< 0.001

*SES-VRQoL* socioemotional scale; reference category for gender is male participants

We additionally stratified the regression models for patients under 65 years of age and participants 65 years and older in two subgroups. Regarding the change in VFS, in both age groups, the visual acuity in the better-/worse-seeing eye was associated with a change in VFS.

The impact of changes in visual acuity on the VFS change was higher in the older age group: a greater reduction in VFS over 5 years due to change in visual acuity was observed for the better- and worse-seeing eye. Baseline socioeconomic status was only associated in the younger group, while change in socioeconomic status was not associated with VFS change (Suppl. Table 3a and Suppl. Table 3b).

Moreover, we stratified the regression models for female and male participants. Female participants showed a greater decrease in VFS scores over 5 years associated with visual acuity change. An association between baseline visual acuity in the worse-seeing eye and change in VFS was also related to female and male participants (Suppl. Table 4a and Suppl. Table 4b). Furthermore, we analyzed the potential interaction between gender and VFS change over 5 years. Univariable analyses showed an association between the interaction factor of gender and 5-year change in visual acuity in the better-seeing and worse-seeing eyes with respect to change in VFS over 5 years (*p* < 0.001), while not in the multivariable model (Suppl. Table 5).

## Discussion

This study investigated VFS and SES-VRQoL change over 5 years and its association concerning age, gender, changes in socioeconomic status, and changes in visual acuity in the better- and worse-seeing eye. Participants who improved from moderate/severe VI to no VI over the 5-year period experienced a substantial improvement in VFS scores (+ 14.76 points). Moreover, the descriptive results demonstrated a small improvement in VFS scores (+ 0.83 points) in participants whose vision worsened from mild VI at baseline to moderate/severe VI in the better-seeing eye at the 5-year follow-up. This finding is unexpected, but it may be explained by psychological adjustment to VI [29]. As participants adjust to their condition over time, their perceived quality of life may stabilize or even improve, despite the objective worsening of their vision. Due to the small number of participants (*n* = 4), this result should be interpreted with caution.

The results demonstrated that changes in visual acuity were associated with VFS change with a higher decrease in older participants and female participants. In general, individuals aged 65 years or older had a smaller decline in VFS over 5 years. This can be explained by the fact that participants of higher age may already have had a decline in VFS in the previous years, which means that it is now less pronounced. Higher baseline socioeconomic status led to a slight positive change in VFS. In the multivariable regression analysis, an association was found regarding visual acuity: a lower baseline visual acuity leads to lower VFS. Moreover, there was a negative association between change in visual acuity in the better- and worse-seeing eye with VFS change. Female participants showed a more significant decrease in VFS scores over 5 years associated with visual acuity change. Socioeconomic status at baseline was positively associated with SES-VRQoL change, but change in socioeconomic status was not associated with SES-VRQoL change over 5 years. Visual acuity in the better-seeing eye at baseline was accompanied by an increase in SES-VRQoL over 5 years. Change in visual acuity in the better- and worse-seeing eye over 5 years was associated with change in SES-VRQoL. Age and gender were not associated with SES-VRQoL change.

A population-based study showed a VFS value of 88.5 for men and 88.1 for women (adjusted for age), in our study the values are slightly higher with 89.59 for men and 89.56 for women at baseline [30]. The findings of this study regarding the change in VFS and change in visual acuity are like those of a longitudinal study examining VFS change over 4 years. The authors reported that decreased visual acuity is associated with decreased VRQoL [19]. The Submacular Surgery Trials Research Group said that NEI-VFQ scores were sensitive to changes in visual acuity (over 2 years). The study demonstrated that a 4-point change in the overall NEI-VFQ score was assessed to be the minimal clinically important difference (MCID) [31]. Several other clinical and population-based studies demonstrated that a decrease in visual acuity or VI leads to changes in VRQoL [1, 4, 32, 33]. In our analysis over 5 years, the change in visual acuity of the worse-seeing eye resulted in a slightly more significant reduction in VRQoL than that of the better-seeing eye. VFS decreased by seven score points in the worse-seeing eye over 5 years per 1 LogMar; while in the group of persons 65 years and older, the reduction in the worse-seeing eye was −13.24.

Cross-sectional studies have reported an association between older age and reduced VRQoL [8]. Our study also indicated that in individuals 65 years of age or older, the reduction in VFS scores due to a change in visual acuity was higher than that in individuals aged < 65 years. In general, the decline in VI is part of the aging process, accompanied by several factors leading to lower visual functioning. Projection of light onto the retina changes with age: biochemical processes alter the cornea, lens opacity, and the ability to accommodate. Furthermore, sensory information processing is impaired. For example, the number of neurons in the ganglion cell layer of the retina and the number of rods in parafoveal vision decrease [34]. Some studies have shown that the typical age-related changes limit mobility and consequently have a negative impact on the quality of life [35–37].

Our findings demonstrate an association between gender and change in VFS. Other studies have also found that female participants were associated with lower VRQoL [38, 39]. There may be several reasons for this finding. One reason could be that women generally have a higher risk of VI in all age groups compared to men [40–42]. Inequalities in diagnosing and treating ophthalmic diseases could be an underlying reason for this. A study in Spain showed that women are less able to pay for private services and, thus, must wait longer for treatment [43]. Biological differences may also be a relevant factor: sex hormones, e.g. due to menopause, may play a role in the prevalence of diseases in ophthalmology, such as glaucoma and age-related macular degeneration [44]. Some mental disorders are more common in women, like depression [45]. This could also be a reason, as they lead to lower self-care, making doctor visits more difficult [46].

Several cross-sectional studies have reported an association between low socioeconomic status and lower VRQoL [20, 47, 48]. This could be based on the fact that a higher level of education leads to a better understanding of eye diseases, earlier consultation of an ophthalmologist in case of symptoms, and different psychological handling of a limitation in vision due to a better understanding [49, 50]. Thus, prevention programs should reach especially people with lower socioeconomic status, which now is the opposite. This is because people with higher educational background tend to think more about their health and, therefore, attend prevention programs more likely [51]. Our study also indicated a slight improvement in VFS and SES-VRQoL scores over 5 years due to a higher socioeconomic status at baseline. Furthermore, an association is also possible, because two integral dimensions of the questionnaire are mental health and social functioning; both dimensions have been associated with socioeconomic status in the past [52–54].

In a clinical setting, the NEI-VFQ-25 questionnaire could be an appropriate method for assessing the success of treatment and surgical interventions. Patients after cataract surgery showed significant improvements of VRQoL [55, 56].

## Strengths and limitations

This study analyzed data from a large population-based representative sample. The GHS is one of the first studies to investigate the change in VRQoL using Rasch-transformed data derived from the NEI-VFQ-25 questionnaire. While previous studies have examined these relationships, the added value of our research lies in its longitudinal design in a large cohort, which tracks changes over a 5-year period. However, our study has some limitations that need to be considered. First, the GHS subjects mainly consist of Caucasian origin. Therefore, the results cannot be generalized to other ethnicities. Second, we utilized a conventional Rasch-based scoring method for the NEI VFQ-25 in this study. The recently developed NEI VFQ-25C approach by Goldstein et al. [57], which applies advanced Rasch analysis for improved precision and comparability in a uni-dimensional scale, could provide additional insights and is worth considering in future studies. Unfortunately, this approach was not feasible in our study, as not all questions required for the NEI VFQ-25C scoring were included in our questionnaire. Third, the number of subjects with VI in the general population is relatively low. Thus, the results show the change on a population-based level rather than in a clinical setting.

## Conclusion

This study supports existing research on the association between visual acuity and VRQoL, with a more pronounced decline observed in females and participants aged 65 years and older. Importantly, by employing a longitudinal approach over a 5-year period, our findings add new insights to the existing body of literature, highlighting the significant impact of both the better-seeing and worse-seeing eyes on changes in VRQoL.

## Supplementary Information

Below is the link to the electronic supplementary material.

Supplementary file1 (DOCX 206 KB)

## References

[1] Yibekal BT, Alemu DS, Anbesse DH et al (2020) Vision-related quality of life among adult patients with visual impairment at University of Gondar, Northwest Ethiopia. J Ophthalmol 2020:9056097. 10.1155/2020/9056097 32280539 10.1155/2020/9056097 PMC7125459

[2] Panigrahi A, Nageswar Rao G, KumariKonar A (2021) Vision-related quality of life and its sociodemographic correlates among individuals with visual impairments. J Vis Impair Blindness 115:319–328. 10.1177/0145482x211028938

[3] Nickels S, Schuster AK, Elflein H et al (2019) Vision-related quality of life considering both eyes: results from the German population-based Gutenberg Health Study (GHS). Health Qual Life Outcomes 17:98. 10.1186/s12955-019-1158-1 31170975 10.1186/s12955-019-1158-1 PMC6554962

[4] Adigun K, Oluleye TS, Ladipo MM et al (2014) Quality of life in patients with visual impairment in Ibadan: a clinical study in primary care. J Multidiscip Healthc 7:173–178. 10.2147/jmdh.S51359 24790455 10.2147/JMDH.S51359 PMC4000176

[5] Finger RP, Fenwick E, Marella M et al (2011) The impact of vision impairment on vision-specific quality of life in Germany. Invest Ophthalmol Vis Sci 52:3613–3619. 10.1167/iovs.10-7127 21357395 10.1167/iovs.10-7127

[6] Parrish RK II, Gedde SJ, Scott IU et al (1997) Visual function and quality of life among patients with glaucoma. Arch Ophthalmol 115:1447–1455. 10.1001/archopht.1997.01100160617016 9366678 10.1001/archopht.1997.01100160617016

[7] Frost A, Eachus J, Sparrow J et al (2001) Vision-related quality of life impairment in an elderly UK population: associations with age, sex, social class and material deprivation. Eye (Lond) 15:739–744. 10.1038/eye.2001.241 11826994 10.1038/eye.2001.241

[8] Man REK, Gan ATL, Fenwick EK et al (2021) The differential impact of age on vision-related quality of life across the visual impairment spectrum. Ophthalmology 128:354–363. 10.1016/j.ophtha.2020.07.046 32738259 10.1016/j.ophtha.2020.07.046

[9] Bertelmann T, Feltgen N, Scheffler M et al (2016) Vision-related quality of life in patients receiving intravitreal ranibizumab injections in routine clinical practice: baseline data from the German OCEAN study. Health Qual Life Outcomes 14:132. 10.1186/s12955-016-0536-1 27644469 10.1186/s12955-016-0536-1 PMC5029004

[10] Nickels S, Schuster AK, Singer S et al (2017) The National Eye Institute 25-Item Visual Function Questionnaire (NEI VFQ-25) - reference data from the German population-based Gutenberg Health Study (GHS). Health Qual Life Outcomes 15:156. 10.1186/s12955-017-0732-7 28789656 10.1186/s12955-017-0732-7 PMC5549396

[11] Hajian-Tilaki K, Heidari B, Hajian-Tilaki A (2017) Are gender differences in health-related quality of life attributable to sociodemographic characteristics and chronic disease conditions in elderly people? Int J Prev Med 8:95. 10.4103/ijpvm.IJPVM_197_16 29184646 10.4103/ijpvm.IJPVM_197_16 PMC5686916

[12] Gallicchio L, Hoffman SC, Helzlsouer KJ (2007) The relationship between gender, social support, and health-related quality of life in a community-based study in Washington County, Maryland. Qual Life Res 16:777–786. 10.1007/s11136-006-9162-4 17286195 10.1007/s11136-006-9162-4

[13] Cherepanov D, Palta M, Fryback DG et al (2010) Gender differences in health-related quality-of-life are partly explained by sociodemographic and socioeconomic variation between adult men and women in the US: evidence from four US nationally representative data sets. Qual Life Res 19:1115–1124. 10.1007/s11136-010-9673-x 20496168 10.1007/s11136-010-9673-x PMC2940034

[14] Jörngården A, Wettergen L, von Essen L (2006) Measuring health-related quality of life in adolescents and young adults: Swedish normative data for the SF-36 and the HADS, and the influence of age, gender, and method of administration. Health Qual Life Outcomes 4:91. 10.1186/1477-7525-4-91 17140436 10.1186/1477-7525-4-91 PMC1697805

[15] Courtright P (2009) Gender and blindness: Taking a global and a local perspective. Oman J Ophthalmol 2:55–56. 10.4103/0974-620x.53032 20671829 10.4103/0974-620X.53032 PMC2905179

[16] Fang R, Yu Y-F, Li E-J et al (2022) Global, regional, national burden and gender disparity of cataract: findings from the global burden of disease study 2019. BMC Public Health 22:2068. 10.1186/s12889-022-14491-0 36369026 10.1186/s12889-022-14491-0 PMC9652134

[17] Barsky AJ, Peekna HM, Borus JF (2001) Somatic symptom reporting in women and men. J Gen Intern Med 16:266–275. 10.1046/j.1525-1497.2001.00229.x 11318929 10.1046/j.1525-1497.2001.00229.x PMC1495200

[18] Harutyunyan T, Giloyan A, Petrosyan V (2017) Factors associated with vision-related quality of life among the adult population living in Nagorno Karabagh. Public Health 153:137–146. 10.1016/j.puhe.2017.09.004 29049920 10.1016/j.puhe.2017.09.004

[19] McKean-Cowdin R, Varma R, Hays RD et al (2010) Longitudinal changes in visual acuity and health-related quality of life: the Los Angeles Latino Eye study. Ophthalmology 117:1900–1907, 1907.e1901. 10.1016/j.ophtha.2010.01.059 20570364 10.1016/j.ophtha.2010.01.059 PMC2945425

[20] Kuo YS, Liu CJ, Cheng HC et al (2017) Impact of socioeconomic status on vision-related quality of life in primary open-angle glaucoma. Eye (Lond) 31:1480–1487. 10.1038/eye.2017.99 28574498 10.1038/eye.2017.99 PMC5639203

[21] Chatziralli IP, Sergentanis TN, Peponis VG et al (2013) Risk factors for poor vision-related quality of life among cataract patients. Evaluation of baseline data. Graefes Arch Clin Exp Ophthalmol 251:783–789. 10.1007/s00417-012-2194-2 23150044 10.1007/s00417-012-2194-2

[22] Mangione CM, Lee PP, Gutierrez PR et al (2001) Development of the 25-item National Eye Institute Visual Function Questionnaire. Arch Ophthalmol 119:1050–1058. 10.1001/archopht.119.7.1050 11448327 10.1001/archopht.119.7.1050

[23] Lampert T, Kroll LE (2009) Die Messung des sozioökonomischen Status in sozialepidemiologischen Studien. In: Richter M, Hurrelmann K (eds) Gesundheitliche Ungleichheit: Grundlagen, Probleme, Perspektiven. VS Verlag für Sozialwissenschaften, Wiesbaden, pp 309–334

[24] Pesudovs K, Gothwal VK, Wright T et al (2010) Remediating serious flaws in the National Eye Institute Visual Function Questionnaire. J Cataract Refract Surg 36:718–732. 10.1016/j.jcrs.2009.11.019 20457362 10.1016/j.jcrs.2009.11.019

[25] Dougherty BE, Bullimore MA (2010) Comparison of scoring approaches for the NEI VFQ-25 in low vision. Optom Vis Sci 87:543–548. 10.1097/OPX.0b013e3181e61bd8 20526224 10.1097/OPX.0b013e3181e61bd8 PMC2924616

[26] Petrillo J, Cano SJ, McLeod LD et al (2015) Using classical test theory, item response theory, and Rasch measurement theory to evaluate patient-reported outcome measures: a comparison of worked examples. Value Health 18:25–34. 10.1016/j.jval.2014.10.005 25595231 10.1016/j.jval.2014.10.005

[27] Rosser DA, Cousens SN, Murdoch IE et al (2003) How sensitive to clinical change are ETDRS logMAR visual acuity measurements? Invest Ophthalmol Vis Sci 44:3278–3281. 10.1167/iovs.02-1100 12882770 10.1167/iovs.02-1100

[28] Coleman AL, Yu F, Ensrud KE et al (2010) Impact of age-related macular degeneration on vision-specific quality of life: Follow-up from the 10-year and 15-year visits of the Study of Osteoporotic Fractures. Am J Ophthalmol 150:683–691. 10.1016/j.ajo.2010.05.030 20691423 10.1016/j.ajo.2010.05.030 PMC2967587

[29] Bergeron CM, Wanet-Defalque M-C (2013) Psychological adaptation to visual impairment: The traditional grief process revised. Br J Vis Impair 31:20–31. 10.1177/0264619612469371

[30] Chia E-M, Mitchell P, Ojaimi E et al (2006) Assessment of vision-related quality of life in an older population subsample: The Blue Mountains Eye Study. Ophthalmic Epidemiol 13:371–377. 10.1080/09286580600864794 17169850 10.1080/09286580600864794

[31] Submacular Surgery Trials Research Group (2007) Evaluation of minimum clinically meaningful changes in scores on the national eye institute visual function questionnaire (NEI-VFQ) SST report number 19. Ophthalmic Epidemiol 14:205–215. 10.1080/09286580701502970 10.1080/09286580701502970 17896299

[32] Sugar EA, Venugopal V, Thorne JE et al (2017) Longitudinal vision-related quality of life for patients with noninfectious uveitis treated with fluocinolone acetonide implant or systemic corticosteroid therapy. Ophthalmology 124:1662–1669. 10.1016/j.ophtha.2017.05.015 28624167 10.1016/j.ophtha.2017.05.015 PMC5651186

[33] Varma R, Wu J, Chong K et al (2006) Impact of severity and bilaterality of visual impairment on health-related quality of life. Ophthalmology 113:1846–1853. 10.1016/j.ophtha.2006.04.028 16889831 10.1016/j.ophtha.2006.04.028

[34] Andersen GJ (2012) Aging and vision: changes in function and performance from optics to perception. Wiley Interdiscip Rev Cogn Sci 3:403–410. 10.1002/wcs.1167 22919436 10.1002/wcs.1167 PMC3424001

[35] Wahl H-W, Schilling O, Oswald F et al (1999) Psychosocial consequences of age-related visual impairment: Comparison with mobility-impaired older adults and long-term outcome. J Gerontol: Ser B 54B:P304–P316. 10.1093/geronb/54B.5.P304 10.1093/geronb/54b.5.p304 10542823

[36] Wahl HW, Heyl V, Oswald F et al (1998) Deteriorating vision in the elderly: double stress? Ophthalmologe 95:389–399. 10.1007/s003470050286 9703717 10.1007/s003470050286

[37] Bergholz R, Dutescu RM, Steinhagen-Thiessen E et al (2019) Ophthalmologic health status of an aging population-data from the Berlin Aging Study II (BASE-II). Graefes Arch Clin Exp Ophthalmol 257:1981–1988. 10.1007/s00417-019-04386-z 31338586 10.1007/s00417-019-04386-z

[38] Rausch-Koster PT, Rennert KN, Heymans MW et al (2022) Predictors of vision-related quality of life in patients with macular oedema receiving intra-vitreal anti-VEGF treatment. Ophthalmic Physiol Opt 42:849–857. 10.1111/opo.12984 35366334 10.1111/opo.12984 PMC9324141

[39] Sturrock BA, Xie J, Holloway EE et al (2015) The influence of coping on vision-related quality of life in patients with low vision: a prospective longitudinal study. Invest Ophthalmol Vis Sci 56:2416–2422. 10.1167/iovs.14-16223 26066595 10.1167/iovs.14-16223

[40] Evans JR, Fletcher AE, Wormald RPL et al (2002) Prevalence of visual impairment in people aged 75 years and older in Britain: results from the MRC trial of assessment and management of older people in the community. Br J Ophthalmol 86:795–800. 10.1136/bjo.86.7.795 12084753 10.1136/bjo.86.7.795 PMC1771210

[41] Abou-Gareeb I, Lewallen S, Bassett K et al (2001) Gender and blindness: a meta-analysis of population-based prevalence surveys. Ophthalmic Epidemiol 8:39–56. 10.1076/opep.8.1.39.1540 11262681 10.1076/opep.8.1.39.1540

[42] Rius Ulldemolins A, Benach J, Guisasola L et al (2018) Why are there gender inequalities in visual impairment? Eur J Pub Health 29:661–666. 10.1093/eurpub/cky245 10.1093/eurpub/cky245 30500932

[43] García-Corchero JD, Jiménez-Rubio D (2022) Waiting times in healthcare: equal treatment for equal need?. Int J Equity Health 21:184. 10.1186/s12939-022-01799-x 10.1186/s12939-022-01799-x PMC9763792 36539735

[44] Korpole NR, Kurada P, Korpole MR (2022) Gender difference in ocular diseases, risk factors and management with specific reference to role of sex steroid hormones. J Midlife Health 13:20–25. 10.4103/jmh.jmh_28_22 35707312 10.4103/jmh.jmh_28_22 PMC9190954

[45] Salk RH, Hyde JS, Abramson LY (2017) Gender differences in depression in representative national samples: meta-analyses of diagnoses and symptoms. Psychol Bull 143(8):783–822. 10.1037/bul0000102 10.1037/bul0000102 PMC5532074 28447828

[46] Chen C, Chen Y, Huang Q et al (2022) Self-care ability of patients with severe mental disorders: based on community patients investigation in Beijing, China. Front Public Health 10:847098. 10.3389/fpubh.2022.847098 35719645 10.3389/fpubh.2022.847098 PMC9198226

[47] Habsyiyah H, Lestari D, Ariawan I et al (2015) Relationship of socioeconomic factors with vision-related quality of life on severe low vision and blind population in Indonesia. Med J Indones 24:245. 10.13181/mji.v24i4.1245

[48] Nutheti R, Shamanna BR, Nirmalan PK et al (2006) Impact of impaired vision and eye disease on quality of life in Andhra Pradesh. Invest Ophthalmol Vis Sci 47:4742–4748. 10.1167/iovs.06-0020 17065482 10.1167/iovs.06-0020

[49] Levinger N, Beykin G, Grunin M et al (2021) Socioeconomic status and visual outcome in patients with neovascular age-related macular degeneration. Eur J Ophthalmol 31:1094–1100. 10.1177/1120672120920783 32363931 10.1177/1120672120920783 PMC8369906

[50] Nesemann JM, Morocho-Alburqueque N, Quincho-Lopez A et al (2023) Association of vision impairment and blindness with socioeconomic status in adults 50 years and older from Alto Amazonas, Peru. Eye 37:434–439. 10.1038/s41433-021-01870-x 35115717 10.1038/s41433-021-01870-x PMC9905540

[51] Pampel FC, Krueger PM, Denney JT (2010) Socioeconomic disparities in health behaviors. Annu Rev Sociol 36:349–370. 10.1146/annurev.soc.012809.102529 21909182 10.1146/annurev.soc.012809.102529 PMC3169799

[52] Zhang Y, Su D, Chen Y et al (2022) Effect of socioeconomic status on the physical and mental health of the elderly: the mediating effect of social participation. BMC Public Health 22:605. 10.1186/s12889-022-13062-7 35351078 10.1186/s12889-022-13062-7 PMC8962021

[53] Li J, Wang J, Li JY et al (2020) How do socioeconomic status relate to social relationships among adolescents: a school-based study in East China. BMC Pediatr 20:271. 10.1186/s12887-020-02175-w 32493261 10.1186/s12887-020-02175-w PMC7268251

[54] Reiss F, Meyrose AK, Otto C et al (2019) Socioeconomic status, stressful life situations and mental health problems in children and adolescents: Results of the German BELLA cohort-study. PLoS One 14:e0213700. 10.1371/journal.pone.0213700 30865713 10.1371/journal.pone.0213700 PMC6415852

[55] Miura G, Baba T, Tatsumi T et al (2021) Effects of cataract surgery on vision-related quality of life in patients with retinitis pigmentosa and the predictive factors of quality of life improvement. Biomed Res Int 2021:3846867. 10.1155/2021/3846867 34552984 10.1155/2021/3846867 PMC8452408

[56] Javed U, McVeigh K, Scott NW et al (2015) Cataract extraction and patient vision-related quality of life: a cohort study. Eye 29:921–925. 10.1038/eye.2015.70 25976642 10.1038/eye.2015.70 PMC4506347

[57] Goldstein JE, Bradley C, Gross AL et al (2022) The NEI VFQ-25C: Calibrating items in the National Eye Institute Visual Function Questionnaire-25 to enable comparison of outcome measures. Transl Vis Sci Technol 11:10. 10.1167/tvst.11.5.10 35543680 10.1167/tvst.11.5.10 PMC9100478

