# Supplementary material for: Gender-specific changes in vision-related quality of life over time – results from the population-based Gutenberg Health Study
Source: Graefes Arch Clin Exp Ophthalmol. 2025 Feb 11;263(7):1825–35. doi: 10.1007/s00417-025-06741-9 (PMC12373681; doi:10.1007/s00417-025-06741-9)
Supplement: Supplementary file 1 — Supplementary file1 (DOCX 206 KB) [file 417_2025_6741_MOESM1_ESM.docx]

**Supplemental files**

**Figure S1** - Changes of visual functioning and changes of visual acuity [LogMar] over five years in the a) better-seeing and b) worse-seeing eye.

**Table S1**- Vision impairment at baseline and 5-year follow up. Data from the German population-based Gutenberg Health Study (2007-2017).

**Table S2** - Outlier’ characteristics and quality of life parameters. Data from the German population-based Gutenberg Health Study (2007-2012).

**Table S3a** - Association analysis between the change in visual functioning scale (VFS) with visual acuity (in the better-/worse-seeing eye) and socioeconomic status over 5 years. Data from the German population-based Gutenberg Health Study (2007-2017). Linear regression analysis, only participants younger than 65 years.

**Table S3b** - Association analysis between the change in visual functioning scale (VFS) with visual acuity (in the better-/worse-seeing eye) and socioeconomic status over 5 years. Data from the German population-based Gutenberg Health Study (2007-2017). Linear regression analysis, only participants 65 years and older.

**Table S4a** - Association analysis between the change in visual functioning scale (VFS) with visual acuity (in the better-/worse-seeing eye) and socioeconomic status over 5 years. Data from the German population-based Gutenberg Health Study (2007-2017). Linear regression analysis, only female participants.

**Table S4b** - Association analysis between the change in visual functioning scale (VFS) with visual acuity (in the better-/worse-seeing eye) and socioeconomic status over 5 years. Data from the German population-based Gutenberg Health Study (2007-2017). Linear regression analysis, only male participants.

**Table S5 -** Association analysis between the change in visual functioning scale (VFS) with visual acuity (in the better-/worse-seeing eye) and socioeconomic status over 5 years. Data from the German population-based Gutenberg Health Study (2007-2017). Linear regression analysis with incorporation of effect modification for gender.

**Figure S1**. Changes of visual functioning and changes of visual acuity [LogMar] over five years in the a) better-seeing and b) worse-seeing eye.


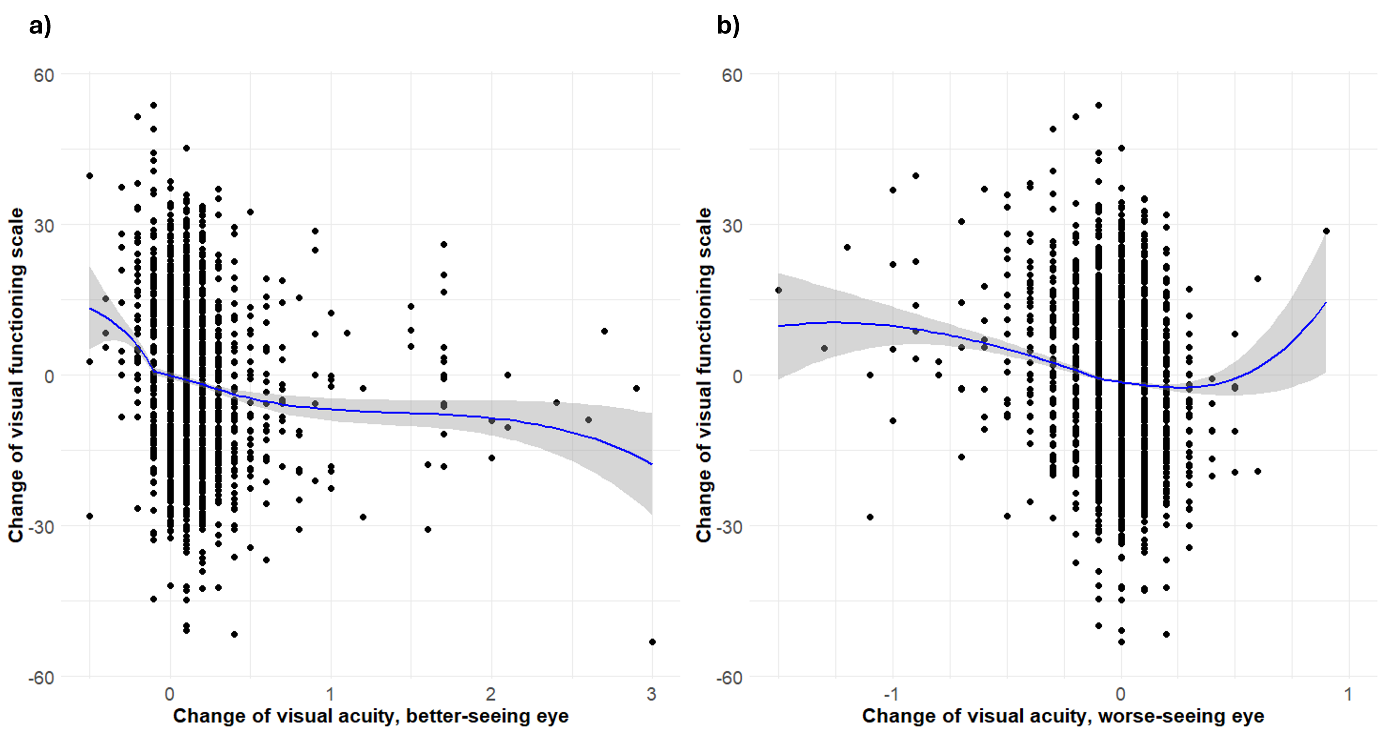


**Table S1**. Vision impairment at baseline and 5-year follow up. Data from the German population-based Gutenberg Health Study (2007-2017).

|  | Worse-seeing eye | | | |
| --- | --- | --- | --- | --- |
|  | **Baseline** | No vision impairment | Mild vision impairment | Moderate/severe vision impairment |
| Better-seeing eye | No vision impairment | 9226 | 184 | 465 |
|  | Mild vision impairment | ­___ | 6 | 22 |
|  | Moderate/severe vision impairment | ___ | ___ | 32 |
|  | **5-year follow-up** | No vision impairment | Mild vision impairment | Moderate/severe vision impairment |
|  | No vision impairment | 8259 | 295 | 443 |
|  | Mild vision impairment | ___ | 32 | 51 |
|  | Moderate/severe vision impairment | ___ | ___ | 51 |

**Table S2**. Outlier’ characteristics and quality of life parameters. Data from the German population-based Gutenberg Health Study (2007-2012).

|  | **Outlier group*** | **Control group** | **p-value** |
| --- | --- | --- | --- |
| **Anthropometric data, baseline** | 118 | 8897 |  |
| Women, n (%) | 77 (65.3) | 4349 (48.9) | 0.001 |
| Age [y], mean (SD) | 51.3 (10.7) | 54.3 (10.8) | 0.003 |
| Age categories, n (%) |  |  | <0.001 |
| 35-44 | 41 (34.7) | 2031 (22.8) |  |
| 45-54 | 41 (34.7) | 2491 (28.0) |  |
| 55-64 | 14 (11.9) | 2419 (27.2) |  |
| 65-74 | 22 (18.6) | 1956 (22.0) |  |
| **Ophthalmic parameters** |  |  |  |
| Visual acuity [logMar], better eye, mean (SD) | 0.04 (0.10) | 0.03 (0.09) | 0.12 |
| Visual acuity [logMar], worse eye, mean (SD) | 0.10 (0.19) | 0.11 (0.22) | 0.81 |
| Spherical equivalent [diopters], right eye, mean (SD) | -0.12 (2.03) | -0.49 (2.48) | 0.11 |
| Spherical equivalent [diopters], left eye, mean (SD) | -0.14 (1.73) | -0.49 (2.51) | 0.13 |
| Glaucoma (ISGEO definition), n (%) | 0 | 64 (0.9) | 0.73 |
| Age-related macular degeneration, n (%) |  |  |  |
| Early AMD, n (%) | 1 (0.85) | 122 (1.37) |  |
| Late AMD, n (%) | 0 | 21 (0.24) |  |
| History of eye surgery, n (%) | 8 (6.8) | 718 (8.1) | 0.99 |
| **General health and socioeconomic status** |  |  |  |
| Arterial hypertension, n (%) | 52 (44.1) | 4196 (47.2) | 0.56 |
| BMI [kg/m²], mean (SD) | 26.54 (5.05) | 27.05 (4.79) | 0.25 |
| Socioeconomic status, mean (SD) | 12.90 (4.24) | 13.47 (4.34) | 0.16 |
| **Vision-related quality of life** |  |  |  |
| Change of visual functioning scale over 5 years | -19.59 (4.17) | -0.85 (9.37) | <0.001 |
| Change of socioemotional scale over 5 years | -5.67 (7.17) | -0.19 (6.79) | <0.001 |
| General health (0-5/best to worst), baseline | 2.10 (0.74) | 2.25 (0.76) | 0.03 |
| General health (0-5/best to worst), follow-up | 2.53 (0.86) | 2.34 (0.81) | <0.001 |

Legend: y – years; SD – standard deviation; AMD – age-related macular degeneration

*The outlier group comprises people whose visual acuity (LogMAR) has changed by less than 0.1 in both eyes over a period of five years, but at the same time have experienced a significant deterioration in visual quality of life (VFS) of more than -15 points. The control group consists of people who also show only a minimal change in visual acuity (less than 0.1 LogMAR) but have not suffered a relevant deterioration in visual quality of life.

**Table S3a**: Association analysis between the change in visual functioning scale (VFS) with visual acuity (in the better-/worse-seeing eye) and socioeconomic status over 5 years. Data from the German population-based Gutenberg Health Study (2007-2017). Linear regression analysis, only participants younger than 65 years.

|  | **Univariable** | | | **Multivariable** | | |
| --- | --- | --- | --- | --- | --- | --- |
| Dependent variable: Change of VFS | B | 95%-CI | p-value | B | 95%-CI | p-value |
| Gender, female | | | | | | |
| Baseline | -0.38 | [-0.79-0.03] | 0.07 | -1.13 | [-1.52- -0.74] | <0.001 |
| VFS | | | | | | |
| Baseline | -0.41 | [-0.43- -0.39] | <0.001 | -0.45 | [-0.47- -0.43] | <0.001 |
| Socioeconomic status | | | | | | |
| Baseline | -0.01 | [-0.06-0.04] | 0.73 | 0.10 | [0.05-0.14] | <0.001 |
| 5-year change | -0.22 | [-0.32- -0.11] | <0.001 | -0.05 | [-0.15-0.05] | 0.31 |
| Visual acuity, better-seeing eye | | | | | | |
| Baseline | 13.86 | [11.18-16.53 | <0.001 | 0.51 | [-2.52-3.55] | 0.74 |
| 5-year change | -4.34 | [-5.84- -2.84] | <0.001 | -4.20 | [-5.72- -2.68] | <0.001 |
| Visual acuity, worse-seeing eye | | | | | | |
| Baseline | 3.15 | [2.17-4.12] | <0.001 | -3.19 | [-4.47- -1.90] | <0.001 |
| 5-year change | -6.46 | [-8.45- -4.47] | <0.001 | -4.17 | [-6.35- -1.99] | <0.001 |

Legend: VFS – Visual functioning scale; reference group for gender is male participants

**Table S3b**: Association analysis between the change in visual functioning scale (VFS) with visual acuity (in the better-/worse-seeing eye) and socioeconomic status over 5 years. Data from the German population-based Gutenberg Health Study (2007-2017). Linear regression analysis, only participants 65 years and older.

|  | **Univariable** | | | **Multivariable** | | |
| --- | --- | --- | --- | --- | --- | --- |
| Dependent variable: Change of VFS | B | 95%-CI | p-value | B | 95%-CI | p-value |
| Gender, female | | | | | | |
| Baseline | -0.31 | [-1.18-0.56] | 0.49 | -0.94 | [-1.78- -0.10] | 0.03 |
| VFS | | | | | | |
| Baseline | -0.43 | [-0.46- -0.39] | <0.001 | -0.44 | [-0.48- -0.41] | <0.001 |
| Socioeconomic status | | | | | | |
| Baseline | -0.04 | [-0.11-0.06] | 0.39 | -0.06 | [-0.15-0.04] | 0.27 |
| 5-year change | -0.14 | [-0.37-0.10] | 0.25 | -0.17 | [-0.38-0.05]] | 0.13 |
| Visual acuity, better-seeing eye | | | | | | |
| Baseline | 11.49 | [7.27-15.71] | <0.001 | -7.67 | [-12.37- -2.96] | 0.001 |
| 5-year change | -9.64 | [-11.99-7.30] | <0.001 | -7.02 | [-9.41- -4.64] | <0.001 |
| Visual acuity, worse-seeing eye | | | | | | |
| Baseline | 3.86 | [2.08-5.64] | <0.001 | -3.46 | [-5.75- -1.18] | 0.003 |
| 5-year change | -19.48 | [-22.48- -16.49] | <0.001 | -13.24 | [-16.70- -9.78] | <0.001 |

Legend: VFS – Visual functioning scale; reference group for gender is male participants

**Table S4a**: Association analysis between the change in visual functioning scale (VFS) with visual acuity (in the better-/worse-seeing eye) and socioeconomic status over 5 years. Data from the German population-based Gutenberg Health Study (2007-2017). Linear regression analysis, only female participants.

|  | **Univariable** | | | **Multivariable** | | |
| --- | --- | --- | --- | --- | --- | --- |
| Dependent variable: Change of VFS | B | 95%-CI | p-value | B | 95%-CI | p-value |
| Age | | | | | | |
| Baseline | 0.07 | [0.04-0.09] | <0.001 | 0.03 | [0.01-0.06] | 0.02 |
| VFS | | | | | | |
| Baseline | -0.42 | [-0.45- -0.40] | <0.001 | -0.45 | [-0.48- -0.43] | <0.001 |
| Socioeconomic status | | | | | | |
| Baseline | -0.04 | [-0.11-0.02] | 0.20 | 0.07 | [-0.00-0.14] | 0.06 |
| 5-year change | -0.13 | [-0.28-0.01] | 0.08 | 0.00 | [-0.14-0.14] | 0.99 |
| Visual acuity, better-seeing eye | | | | | | |
| Baseline | 13.37 | [10.32-16.42] | <0.001 | -2.89 | [-6.58-0.80] | 0.12 |
| 5-year change | -7.67 | [-9.51- -5.83] | <0.001 | -6.12 | [-8.00- -4.24] | <0.001 |
| Visual acuity, worse-seeing eye | | | | | | |
| Baseline | 3.84 | [2.58-5.10] | <0.001 | -3.34 | [-5.03- -1.65] | <0.001 |
| 5-year change | -13.58 | [-16.09- -11.07] | <0.001 | -8.84 | [-11.60- -6.09] | <0.001 |

Legend: VFS – Visual functioning scale

**Table S4b**: Association analysis between the change in visual functioning scale (VFS) with visual acuity (in the better-/worse-seeing eye) and socioeconomic status over 5 years. Data from the German population-based Gutenberg Health Study (2007-2017). Linear regression analysis, only male participants.

|  | **Univariable** | | | **Multivariable** | | |
| --- | --- | --- | --- | --- | --- | --- |
| Dependent variable: Change of VFS | B | 95%-CI | p-value | B | 95%-CI | p-value |
| Age | | | | | | |
| Baseline | 0.07 | [0.05-0.09] | <0.001 | 0.02 | [-0.00-0.05] | 0.05 |
| VFS | | | | | | |
| Baseline | -0.41 | [-0.44- -0.39] | <0.001 | -0.45 | [-0.47- -0.42] | <0.001 |
| Socioeconomic status | | | | | | |
| Baseline | 0.00 | [-0.06-0.06] | 0.99 | 0.07 | [0.02-0.13] | 0.01 |
| 5-year change | -0.26 | [-0.39- -0.13] | <0.001 | -0.11 | [-0.24-0.01] | 0.07 |
| Visual acuity, better-seeing eye | | | | | | |
| Baseline | 9.64 | [6.77-12.51] | <0.001 | -3.50 | [-6.96- -0.03] | 0.05 |
| 5-year change | -4.17 | [-5.85- -2.50] | <0.001 | -4.61 | [-6.34- -2.87] | <0.001 |
| Visual acuity, worse-seeing eye | | | | | | |
| Baseline | 2.67 | [1.56-3.77] | <0.001 | -3.44 | [-4.91- -1.97] | <0.001 |
| 5-year change | -8.29 | [-10.46- -6.13] | <0.001 | -4.61 | [-8.43- -3.57] | <0.001 |

Legend: VFS – Visual functioning scale

**Table S5:** Association analysis between the change in visual functioning scale (VFS) with visual acuity (in the better-/worse-seeing eye) and socioeconomic status over 5 years. Data from the German population-based Gutenberg Health Study (2007-2017). Linear regression analysis with incorporation of effect modification for gender.

|  | **Univariable** | | | **Multivariable** | | |
| --- | --- | --- | --- | --- | --- | --- |
| Dependent variable: Change of VFS | B | 95%-CI | p-value | B | 95%-CI | p-value |
| **Gender, female** | | | | | | |
| Baseline | -0.37 | [-0.74-0.01] | 0.05 | -0.80 | [-4.96-3.35] | 0.70 |
| **Age** | | | | | | |
| Baseline | 0.07 | [0.05-0.09] | <0.001 | 0.02 | [-0.00-0.05] | 0.07 |
| Baseline*sex | -0.00 | [-0.04-0.03] | 0.87 | 0.01 | [-0.03-0.05] | 0.59 |
| **VFS** |  | | | | | |
| Baseline | -0.41 | [-0.43- -0.40] | <0.001 | -0.45 | [-0.47- -0.42] | <0.001 |
| Baseline*sex | -0.01 | [-0.04-0.02] | 0.47 | -0.006 | [-0.04-0.03] | 0.73 |
| **Socioeconomic status** | | | | | | |
| Baseline | 0.00 | [-0.06-0.06] | 0.99 | 0.07 | [0.01-0.13] | 0.02 |
| Baseline*sex | -0.05 | [-0.13-0.04] | 0.31 | -0.006 | [-0.24-0.02] | 0.89 |
| 5-year change | -0.26 | [-0.39- -0.13] | <0.001 | -0.11 | [-0.24-0.02] | 0.09 |
| 5-year change*sex | 0.13 | [-0.06-0.32] | 0.19 | -0.11 | [-0.07-0.30] | 0.24 |
| **Visual acuity, better-seeing eye** | | | | | | |
| Baseline | 9.64 | [6.60-12.68] | <0.001 | -3.50 | [-7.17-0.17] | 0.06 |
| Baseline*sex | 3.73 | [-0.47-7.93] | 0.08 | 0.60 | [-4.47-5.67] | 0.82 |
| 5-year change | -4.17 | [-5.95- -2.39] | <0.001 | -4.61 | [-6.44- -2.77] | <0.001 |
| 5-year change*sex | -3.50 | [-5.99- -1.01] | 0.006 | -1.52 | [-4.08-1.04] | 0.25 |
| **Visual acuity, worse-seeing eye** | | | | | | |
| Baseline | 2.67 | [1.49-3.84] | <0.001 | -3.44 | [-4.99- -1.89] | <0.001 |
| Baseline*sex | 1.17 | [-0.49-2.84] | 0.17 | 0.09 | [-2.14-2.33] | 0.93 |
| 5-year change | -8.29 | [-10.59- -6.00] | <0.001 | -5.99 | [-8.57- -3.43] | <0.001 |
| 5-year change*sex | -5.29 | [-8.59- -1.98] | 0.002 | -2.84 | [-6.51-0.82] | 0.13 |

Legend: VFS – Visual functioning scale; Adjustments were made for the effect of gender in each univariable analysis. Univariable interaction model: Visual functioning scale ~ gender+predictor+predictor*gender
